# Effect of PGY Training Year on Perceived Readiness to Perform Entrustable Professional Activities

**DOI:** 10.15694/mep.2017.000181

**Published:** 2017-10-11

**Authors:** William Murdoch, Nicholas Pryomski, Nancy Delaney, Susan Hulsemann

**Affiliations:** 1 ProMedica Monroe Regional Hospital

**Keywords:** Clinical competence, Entrustable Professional Activity, Internship and residency, Accreditation

## Abstract

This article was migrated. The article was marked as recommended.

Background

The Family Medicine for America’s Health (FMAH) collaborative approved a set of Entrustable Professional Activities (EPAs) for the specialty, designed to serve as a set of activities that all practitioners in the specialty can be expected to perform. The extent to which family medicine residents feel equipped to perform EPAs is not known.

Objective

To assess the extent to which family medicine residents in all three PGY years feel ready to perform Entrustable Professional Activities.

Methods

This spring, eighteen residents were asked to complete the “ProMedica Monroe Family Medicine Entrustable Professional Activities Survey,” which asks residents, for all 20 EPAs, to 1) identify their readiness to perform the EPA on a 9-point Likert scale, and 2) endorse their level of PGY training.

Results

Fifteen of out eighteen eligible participants completed the survey. Mean readiness levels across EPAs were 5.23/9 for PGY1s, 7.27 for PGY2s, and 8.17 for PGY3s. Residents reported higher readiness levels with inpatient care (7.67) and relationship building (7.80), but lower levels with mental health care (5.87) and obstetrical care (5.07).

Conclusions

Residents perceive increases in readiness to perform family medicine EPAs with each PGY year. Resident readiness levels are broadly similar across EPAs, with few outliers.

## Introduction

The objective evaluation of resident competency is a challenge for residency programs, faculty, and residents themselves as they develop specific skills that they should possess upon completion of their program. In 2001 the ACGME launched the six Core Competencies with the hope that these would “be an antidote to overspecification of accreditation standards, and that they would empower programs to create training programs grounded in meaningful outcomes in a developmental approach.” ^1^

In 2013, the ACGME implemented the Milestones initiative with the goal of standardizing programs nationwide within their specialty. The Core Competencies themselves are used as a framework to describe the qualities of healthcare professionals in general. ^2^ Entrustable Professional Activities (EPAs) are more specific, practice-based abilities that have been implemented by several medical specialties to help bridge this gap between the generalized professionalism of the competencies and practicing medicine in the healthcare environment of today.

Recently the Family Medicine for America’s Health (FMAH) collaborative conceived and approved a set of EPAs for the specialty. The intent is that all practitioners of Family Medicine will be competent and confident in their ability to perform these skills after residency. This set was created with input from several Family Medicine societies across North America, though at this time the extent to which family medicine residents feel equipped to perform these EPAs is not known.

A more recent recommendation about the practical use of EPAs has been proposed by the Association of Family Medicine Residency Directors (AFMRD). ^3^ Essentially, the AFMRD group acknowledged the resistance to having one more evaluative layer to measure and report on. They proposed, instead, three ways that the EPAs could add value to Family Medicine Programs: 1) improve resident evaluation; 2) serve as curricular evaluation tools and 3) represent concrete descriptors of expectations of resident performance.

While the theoretical and foundational attributes of EPAs are maturing, research exploring the extent to which family medicine residents feel equipped to perform these EPAs has not been developed. The goal of this study is to create a model to assess residents’ comfort and perceived level of competence around each of the twenty Family Medicine EPAs.

## Methods

Eighteen residents at the ProMedica Monroe Family Medicine residency were asked to complete the “ProMedica Monroe EPA Survey,” shown in the next column, which asked residents, for all 20 EPAs, to 1) identify their readiness to perform the EPA on a 9-point Likert scale, and 2) endorse their level of PGY training. All residents were asked to participate in this voluntary survey during protected time; there were no exclusion criteria.

The survey was distributed and collected by research-trained faculty. The results of the study were not intended to be used for individual resident evaluation. Group completion of the survey took approximately fifteen minutes. No identifying information was collected, and the data was analyzed in aggregate form using Microsoft Excel. The study was approved by the ProMedica Health System Institutional Review Board (IRB No. #16-022).

## Results

Fifteen residents at the ProMedica Monroe Family Medicine Residency chose to complete the voluntary survey, out of eighteen eligible (83%). Five respondents each endorsed PGY-1, -2, and -3 training status. All items were answered by all fifteen respondents. Table 1 describes the mean and standard deviation across all EPAs, as experienced by each PGY class, on the 1-9 Likert scale.

Self-reported readiness to perform EPAs increased with each PGY level, with standard deviations suggesting a statistically significant advancement. Table 2 describes the mean, standard deviation, and range for each EPA from 1-9, broken down by PGY class. Individual EPAs are paraphrased in summary form for brevity.

Residents had generally similar readiness levels with all EPAs, with an overall mean of 6.89 (±0.62). Residents reported higher overall readiness levels with inpatient care (7.67, ±1.29) and relationship building (7.80, ±1.22), but less readiness with mental health care (5.87, ±1.53) and obstetrical care (5.07, ±1.55). The greatest increases in readiness appear to occur between PGY-1 and PGY-2. More uniformity is seen across EPAs in the PGY-3 class as compared to junior classes.

## Discussion

Entrustable professional activities define units of work, clinical skills and abilities that family medicine residents should be able to perform as they move through their training. They are standardized targets that are measurable and against which residents and faculty alike should be able to recognize proficiency once achieved.

In our survey, residents were presented with the list of EPAs for Family Medicine. They reported most readiness to perform inpatient based care and relationship building, and felt least ready in the realm of obstetrics and mental health. As anticipated, readiness to perform EPAs increased with PGY level and the lowest and more varied scores were in the PGY-1 class. Entrustment decisions are affected by several variables, and in this study, we examined the responses by level of training.

Using EPAs to improve resident evaluations, improve curricular evaluation tools, and provide concrete descriptors of expectations of resident performance, as recommended by the AFMRD, can assist faculty, residents and programs to focus on quality care in today’s healthcare environment. This integration of EPA language into a broader curricular and evaluative framework is likely to continue at the accreditation level as well. ^4^

Limitations of the study include small sample size and participation at a single residency site, consistent with a pilot study. Future research should certainly include the replication of these findings beyond a pilot population, and expand upon the predictive value of comfort with individual EPAs on choice of practice settings. In addition, research and study focused around on-going resident self-evaluation and feedback from patients related to Family Medicine EPAs can enhance learning by keeping objective, relevant and timely feedback in focus.

## Conclusions

Residents perceive increases in readiness to perform family medicine EPAs with each PGY year. Resident readiness levels are broadly similar across EPAs, with few outliers. Future and ongoing research will include the verification of these findings across residency programs and successive resident cohorts. The assessment and tracking of resident preparedness for EPA performance will be increasingly important as accreditation bodies continue to incorporate EPA and milestone language.

## Notes On Contributors

William Murdoch, MD, FAAFP: Associate Residency Director at PMFMR. Program Director of Traditional Rotating Internship at same site. Lead author and investigator. Clinical focus is teaching inpatient medicine to interns and residents in Family Medicine and Emergency Medicine.

Nicholas Pryomski, MD: Former (now graduated) resident physician at PMFMR. Lead resident on project. Assisted in data collection, literature review, poster preparation, and poster presentation. Now in outpatient primary care practice in Wisconsin.

Nancy Delaney, PhD: Psychologist and behavioral scientist at PMFMR. Participated in data collection, analysis, and manuscript preparation. Leads communications and didactics curricula.

Susan Hulsemann, MD: Residency Director of PMFMR. Participated in manuscript preparation, editing, and project support. Over 15 years of Family Medicine GME experience. Also maintains a private practice.

## Appendices

**Table 1.** Mean and standard deviations of EPA readiness by PGY year

PGY class	Mean	Std. Dev.
1	5.23	0.68
2	7.27	0.91
3	8.17	0.47

**Table 2.** Mean and standard deviations of readiness to perform each EPA by PGY year

EPA	PGY-1	PGY-2	PGY-3
Usual source of comprehensive care	4.6 (0.89)	7.6 (0.89)	8.0 (0.71)
Provide care in multiple settings	5.6 (0.89)	7.8 (0.84)	8.2 (0.84)
First-contact access to care	5.2 (0.45)	7.2 (0.84)	8.8 (0.45)
Provide preventive care	5.4 (0.55)	7.8 (1.10)	8.4 (0.89)
Provide care that speeds recovery	5.6 (0.55)	7.2 (0.45)	8.2 (0.45)
Evaluate undifferentiated symptoms	4.6 (0.89)	7.2 (1.30)	8.4 (0.56)
Manage chronic medical conditions	5.6 (0.55)	7.2 (1.48)	8.4 (0.56)
Manage mental health conditions	4.2 (1.64)	6.2 (0.84)	7.2 (0.45)
Manage acute illness and injury	5.0 (1.22)	7.4 (1.14)	8.2 (0.45)
Perform common procedures	4.6 (1.14)	6.2 (1.64)	8.6 (0.56)
Manage full-spectrum obstetrical care	3.8 (1.92)	4.6 (2.61)	6.8 (1.10)
Manage end-of-life and palliative care	4.8 (2.77)	6.0 (2.24)	8.0 (0.71)
Manage inpatient care and transitions	6.2 (0.84)	8.2 (0.84)	8.6 (0.55)
Manage care for medical emergencies	5.0 (1.22)	7.0 (1.87)	8.0 (0.71)
Develop trusting relationships	6.4 (1.52)	8.4 (0.89)	8.6 (0.55)
Use data to optimize care	5.2 (2.68)	7.8 (1.64)	8.0 (1.22)
Provide care in the context of health beliefs	6.2 (0.84)	7.8 (1.64)	8.2 (0.84)
Advocate for patients and communities	5.8 (1.79)	8.0 (1.41)	8.2 (1.30)
Provide leadership for health care teams	5.0 (1.58)	7.8 (1.30)	8.6 (0.55)
Coordinate care and consultations	5.8 (0.84)	8.0 (0.71)	8.0 (0.71)
