# Supplementary material for: Effect of PGY Training Year on Perceived Readiness to Perform Entrustable Professional Activities
Source: MedEdPublish (2016). 2017 Oct 11;6:181. [Version 1] doi: 10.15694/mep.2017.000181 (PMC10885303; doi:10.15694/mep.2017.000181)
Supplement: Peer review history

# Reviewer report: Reviewer response for version 1

**DOI:** 10.21956/mep.19367.r28340

**Published:** 2017-10-19

**Authors:** Rehan Ahmed Khan

**Affiliations:** 1 Riphah International University

This review has been migrated. The reviewer awarded 3 stars out of 5 I think this is an interesting article. EPA's are entrustment decisions made by supervisors , however to know the readiness of residents prior to an entrustment decision will provide them an opportunity of self evaluation. However it would be interesting to know the co relation between EPA readiness and eventual EPA assessment.The limitations have already been highlighted by the authors as small sample size and single institution.

Reviewer Expertise:

NA

No decision status is available

# Reviewer report: Reviewer response for version 1

**DOI:** 10.21956/mep.19367.r28341

**Published:** 2017-10-18

**Authors:** Alan Jaap

**Affiliations:** 1 University of Edinburgh Medical School

This review has been migrated. The reviewer awarded 3 stars out of 5 This is an interesting pilot study in the emerging field of EPA implementation. I disagree with the comments of some of the other reviewers in that user involvement has not been a prominent feature in the published literature on EPA development and so it is useful to have the trainee perspective, as reported in this study, to provide a reality check about the feasibility and practicality of proposed EPAs.I encourage the authors to continue to pursue this work beyond the pilot stage.

Reviewer Expertise:

NA

No decision status is available

# Reviewer report: Reviewer response for version 1

**DOI:** 10.21956/mep.19367.r28338

**Published:** 2017-10-15

**Authors:** Megan Anakin

**Affiliations:** 1 University of Otago

This review has been migrated. The reviewer awarded 3 stars out of 5 This well-written article provides useful information about graduate student perceptions of their confidence to complete entrustable professional activities. Researchers and practitioners in health professional education will find this article thought-provoking because they may find it helpful to illuminate discussions about entrustable professional activities that pertain to their own particular profession. Aside from the limitations mentioned by the previous two reviewers, this study may provide the groundwork for a follow up study with greater numbers of students or invite a more in-depth study of students’ experiences.

Reviewer Expertise:

NA

No decision status is available

# Reviewer report: Reviewer response for version 1

**DOI:** 10.21956/mep.19367.r28339

**Published:** 2017-10-11

**Authors:** John Cookson

**Affiliations:** 1 University of Worcester

This review has been migrated. The reviewer awarded 3 stars out of 5 Thank you, an interesting paper.However I also have a problem with the self-evaluation. The 'entrustable' in EPAs refers to the supervisor being able to progressively trust the trainee with the activity. The view of the trainee is in a sense redundent (though it's certainly interesting to know). Then, of course, there is the difference between 'confidence' and 'competence'. There is good evidence that good trainees unvervalue themselves and poorer students the reverse.

Reviewer Expertise:

NA

No decision status is available

# Reviewer report: Reviewer response for version 1

**DOI:** 10.21956/mep.19367.r28342

**Published:** 2017-10-11

**Authors:** Trevor Gibbs

**Affiliations:** 1 AMEE

This review has been migrated. The reviewer awarded 4 stars out of 5 An interesting paper that covers a very important area. It was good to see that the scores of the different years of students increased year on year, reflecting some quality upon the curriculum. I was however a little confused about the use of a self- evaluation questionnaire looking at EPAs, which I feel are related exactly to the ability to carry out a specific skill without supervision, or at least degrees up to that level. This I believe grows from a purposeful observation and feedback activity- I wasn't sure if this was present in the learning activities.I do however value this paper and would encourage the authors to consider a more qualitative piece of research that investigates the students perceptions to a greater degree.

Reviewer Expertise:

NA

No decision status is available
